# Is Avoidable Hospitalization Experienced Prior to Infection Associated With COVID-19-Related Deaths?

**DOI:** 10.3389/ijph.2022.1604426

**Published:** 2022-06-20

**Authors:** Woo-Ri Lee, Ki-Bong Yoo, Gyeong-Min Lee, Jun Hyuk Koo, Li-Hyun Kim

**Affiliations:** ^1^ Department of Health Administration, Yonsei University, Wonju, South Korea; ^2^ Yonsei University Wonju Industry-Academic Cooperation Foundation, Wonju, South Korea

**Keywords:** COVID-19, primary health care, ACSC, NHIS-COVID-19 data, avoidable hospitalization, underlying disease, continuity of care, DRE

## Abstract

**Objectives:** This study aimed to determine the effect of the presence or absence of avoidable hospitalization before acquiring coronavirus disease (COVID-19) on COVID-19-related deaths.

**Methods:** This study used the total NHIS-COVID-19 dataset comprising domestic COVID-19 patients, provided by the National Health Insurance Service (NHIS) in South Korea. We conducted logistic regression and double robust estimation (DRE) to confirm the effect of avoidable hospitalization on COVID-19-related deaths.

**Results:** Logistic regression analysis confirmed that the odds ratio (OR) of death due to COVID-19 was high in the group that experienced avoidable hospitalization. DRE analysis showed a higher OR of death due to COVID-19 in the group that experienced avoidable hospitalization compared to the group that did not experience avoidable hospitalization, except in the subgroup aged ≤69 years.

**Conclusion:** The effect of avoidable hospitalization on COVID-19-related deaths was confirmed. Therefore, continued health care, preventive medicine, and public health management are essential for reducing avoidable hospitalizations despite the COVID-19 pandemic. Clinicians need to be informed about the importance of continuous disease management.

## Introduction

In December 2019, an unexplained case of pneumonia was reported in Wuhan, China, which soon was shown to be a new type of coronavirus that was transmitted to humans [1]. Subsequently, when the virus spread worldwide, on 11 March 2020, the World Health Organization declared the third pandemic in history [2]. The first case in South Korea was confirmed on 21 January 2020, and the first death occurred on 20 February 2020 [3]. On 18 February 2020, the number of confirmed coronavirus disease 2019 (COVID-19) cases and COVID-19 deaths increased sharply [4]. Consequently, the government implemented a social distancing policy to prevent the spread of COVID-19 and proceeded with social distancing in two stages. Stage 1 involved wearing masks and following personal hygiene and disinfection guidelines. At the same time, it was recommended to refrain from operating facilities with a high risk of outbreaks (religious facilities, indoor physical education facilities, clubs, pubs, private educational institutes, etc.). Stage 2 closed down the operation of facilities at high risk of outbreaks in Stage 1 (Supplementary Figure S1).

In view of the COVID-19 pandemic, numerous studies related to infections and deaths due to COVID-19 have been conducted. Most studies have shown that elderly people, especially those with underlying diseases, are vulnerable to contracting COVID-19 because they have a relatively weak immune system [5, 6] and are at a higher risk of death due to diabetes, high blood pressure, obesity, coronary heart disease, and chronic obstructive pulmonary disease (COPD) [7–11]. Additionally, the high number of underlying diseases tends to increase COVID-19 severity [12, 13], which suggests that underlying diseases are a critical variable during the ongoing COVID-19 pandemic.

Ambulatory care-sensitive conditions (ACSC) can prevent disease aggravation and avoid unnecessary hospitalization through appropriate outpatient services [14]. ACSCs are classified according to Billings et al. [14]. Hospitalization for ACSCs is called avoidable hospitalization, which means unnecessary hospitalization due to the lack of appropriate outpatient treatment. Experiencing avoidable hospitalizations is regarded as an indicator closely related to the quality of primary care and healthcare access [15, 16]. In other words, experiencing avoidable hospitalizations reflects improper functioning of the primary healthcare system due to medical system-related reasons (absence of an effective system) or patients’ personal reasons (such as economic reasons or lack of continuity of care). This also means that patients do not receive any ongoing management.

ACSCs are mostly chronic diseases; therefore, it is crucial to prevent escalation of the disease through systematic management so that it does not lead to hospitalization. Williamson et al. confirmed that underlying diseases, such as obesity, diabetes, cancer, hematological malignancy, asthma, and chronic respiratory/heart/liver diseases, are risk factors for death due to COVID-19 [17].

As the paradigm shifts from disease treatment-oriented medicine to prevention-and management-oriented medicine, the importance of continuous and effective management of underlying diseases has been emphasized. Recently, outpatient treatment has become paralyzed owing to the difficulty in visiting hospitals because of the COVID-19 crisis, but disease management is essential even in this situation [18]. The aim of this study is to determine the effect of the presence or absence of avoidable hospitalizations before infection or death due to COVID-19 in patients with underlying diseases.

## Methods

### COVID-19 Testing Strategy

Following the guidelines of the Korea Centers for Disease Control and Prevention (KCDC) [19], tests were conducted on pseudo-patients and symptomatic patients. COVID-19 was confirmed by polymerase chain reaction (PCR), and the test targets were as follows: those who developed a fever (>37.5°C) or respiratory symptoms (cough, dyspnea, other symptoms) within 14 days of contact with a confirmed COVID-19 patient; those who had visited overseas and developed a fever (>37.5°C) or respiratory symptoms within 14 days of returning to Korea; those who, according to a doctor’s opinion, were suspected of being infected with COVID-19, such as pneumonia of unknown cause; those who were epidemiologically related to the COVID-19 outbreak in Korea and had a fever (>37.5°C) or respiratory symptoms within 14 days.

In the case of pseudo-patients and symptomatic patients, specimens were collected by moving the patient to an isolated or independent space prepared in a triage room (public health center or drive-through, walk-through diagnostic test). If the test result was negative, quarantine was lifted after the self-isolation period (14 days as of the final contact date) was maintained. If the test result was positive, the patient was hospitalized based on symptom severity.

Because all pseudo-patients and symptomatic patients in South Korea were tested according to the test strategy, the possibility of detection bias was low.

### Data

The National Health Insurance Service COVID-19 data (NHIS-COVID-19 DATA) were used for the analysis. The NHIS-COVID-19 data combine data from the NHIS and Korea Disease Control and Prevention Agency (KCDC) for COVID-19-related research. Claims data provided by the NHIS and KCDC COVID-19 screening results, treatment results, and demographic information were combined to provide the data. The NHIS-COVID-19 data consisted of the control population, test-negative group, and COVID-19 patient group. For the control population, 15 times the number of COVID-19 patients were extracted by stratification according to sex, age, and region among COVID-19 patients and the general population who did not test negative. In the test-negative group, the patients were classified under a COVID-19-related disease code, but the PCR result was negative. In the case of a COVID-19 patient, the PCR result was positive, and it was claimed as U071 and U072 (diagnostic codes related to COVID-19 based on the ICD-10 code). Data between 1 January 2019, and 14 August 2020, were used. The control population and those who tested negative were excluded from the study because they were inappropriate for study purposes [20, 21]. Among the 8,080 confirmed cases of COVID-19, 7,953 were included in the study, and 127 people with unclear insurance types were excluded (Supplementary Figure S2). All analyses were performed using SAS, version 9.4 (SAS Institute, Cary, NC, United States). The study was approved by the institutional review board (1041849-202007-SB-088-01).

### Study Variables

#### Outcome Variables

In the case of the COVID-19 patient group, based on the ICD-10 code, they were charged with the claim codes “U071” that corresponded to confirmed cases of COVID-19, and the PCR result was positive. Among the COVID-19 patient group, COVID-19-related deaths were coded as 1, and survivors were coded as 0.

#### Exposure Variables

According to a previous study, we classified patients hospitalized for ACSCs suitable for the Korean situation [22] as “1” and those who did not as “0.” We included subjects with meningococcal infection (A39); emphysema (J43); meningitis in bacterial diseases classified elsewhere (G01); bacterial meningitis, not elsewhere classified (G00); nondiabetic hypoglycemic coma (E15); other disorders of fluid, electrolyte, and acid–base balance (E87); convulsions, not elsewhere classified (R56); other disorders of pancreatic internal secretion (E16); volume depletion (E86); other acute ischemic heart diseases (I24); other respiratory disorders (J98); pneumonia (J09-J18); unspecified diabetes mellitus (E14); fever of other and unknown origin (R50); dizziness and giddiness (R42); angina pectoris (I20); essential hypertension (I10); other noninfectious gastroenteritis and colitis (K52); influenza, virus not identified (J11); cutaneous abscess, furuncle, and carbuncle (L02); acute tonsillitis (J03); acute upper respiratory infections (J00, J01, J02, J04, and J05); acute upper respiratory infections of multiple and unspecified sites (J06); and chronic rhinitis, nasopharyngitis, and pharyngitis (J31).

#### Confounding Variables

Sex, age, and insurance type were included as individual characteristics, and disability and the number of underlying diseases were considered health-related characteristics. The COVID-19 fatality rate in Korea was confirmed to exceed 1% among those aged 70 years or older [23]. Accordingly, age was stratified as ≤ 69 and ≥ 70 years. The medical insurance type was stratified into national health insurance and medical aid which is provided to low-income individuals [24]. In the case of disability grade, according to the Korean disability classification system, the disability grade is divided into grades 1 to 6, with 1–2 being severe, 3–6 being mild, and others non-disabled [25]. As for underlying diseases, cancer (C00-C97), hypertension (I10 and I15), diabetes (E10, E11, E13, and E14), hypercholesterolemia (E78), myocardial infarction (I21 and I22), stroke (I60-I63), heart failure (I50), asthma (J45 and J46), COPD (J44), chronic kidney disease (N18), chronic hepatitis B (B180, B181, and B1810), and chronic hepatitis C (B182) were included based on the ICD-10-CM standard as described in a previous study [10]. Underlying diseases were classified as no disease and one, two, three, or more diseases.

### Study Design

The causal relationship between variables was expressed using a directed acyclic graph (DAG) (Supplementary Figure S3). When examining the relationship between variables in the DAG, it can be seen that the variables used as control variables in this study, such as sex, age, insurance type, underlying disease, and disability, affect avoidable hospitalization, which is an exposure variable. The relationship between these variables was based on the Andersen model, a representative model for predicting an individual’s use of medical services. The Andersen model is divided into predisposing, enabling, and needs factors [26, 27]. The predisposing factor refers to a given factor, regardless of an individual’s will, and includes sex and age. Enabling factors refer to financial resources and environmental factors that make medical use possible, including the insurance type. The needs factor refers to a disease or health condition that acts as a direct determinant of the use of medical services, and includes disabilities and underlying diseases. Based on the Andersen model, three factors can explain the relationship with avoidable hospitalization. In the case of age, sex, underlying disease, and disability, we confirmed a significant relationship in previous studies on COVID-19-related deaths and established the relationship accordingly [5, 10, 28, 29].

### Statistical Analysis

Data were analyzed using binary logistic regression and the doubly robust estimation (DRE) method using inverse probability treatment weighting (IPTW). IPTW was calculated as the reciprocal of the propensity score (PS). DRE is a causal relationship estimation method that adjusts the covariates used to calculate PS once more by including them in the final analysis model [30, 31]. This allowed us to estimate the Average Treatment Effect (ATE). In the analysis, age, sex, insurance type, underlying disease, and disability variables were adjusted to control for the effect of covariates, excluding avoidable hospitalization on COVID-19-related deaths.

Subgroup analysis was carried out using logistic regression analysis and DRE analysis, divided into cases ≤ 69 years of age, ≤ 70 years of age, and underlying diseases. In the case of age groups, it was necessary to stratify and analyze because the difference in fatality rate was significantly related to age. In the case of the underlying disease group, it was necessary to stratify the analysis because the fatality rate was higher in those with underlying diseases. The analysis was conducted with the hypothesis that exposure factors would have a more significant effect on the outcomes in the ≥ 70 years group and the group with underlying diseases compared to the analysis results of the entire group.

## Results

Table 1 presents the cross-tabulation of avoidable hospitalizations and COVID-19-related deaths. Of the 233 COVID-19-related deaths, 27 patients (11.6%) died due to avoidable hospitalization.

**TABLE 1:** Crosstabulation of avoidable hospitalizations and COVID-19-related death (South Korea, 2019–2020).

Variable	Classification	COVID-19-related deaths	
		Yes	No
		n (%)	n (%)
Avoidable hospitalizations in 2019	Yes	27 (11.6)	139 (1.8)
	No	206 (88.4)	7,581 (98.2)
	Total	233 (100.0)	7,720 (100.0)

COVID-19, coronavirus-19 disease.

Table 2 presents the general characteristics of the participants. Of the 7,953 patients, 166 experienced avoidable hospitalization in 2019. The COVID-19 mortality rate among those who experienced avoidable hospitalization was 16.3%, which was approximately seven times higher than that among those who did not experience avoidable hospitalization (2.6%). The study included 3,185 men and 4,768 women, and the COVID-19-related death rate was higher among men than among women. Moreover, 6,941 patients were aged ≤ 69 years and 1,012 people were aged ≥ 70 years. The COVID-19 mortality rate among those aged ≤ 69 years was 0.7% and that among those aged ≥ 70 years was 17.9%. By insurance type, 676 people received medical aid and 7,277 patients had health insurance. The COVID-19 mortality rate among medical aid patients was 6.2% and that among health insurance patients was 2.6%. In terms of disability grade, 7,337 people were non-disabled, and 616 people had severe or mild disabilities. Regarding underlying diseases, 5,067 participants (63.7%) had at least one underlying disease. It was found that the more severe the disability grade, the greater the number of underlying diseases and the higher the COVID-19 mortality rate.

**TABLE 2:** General characteristics of patients (South Korea, 2019–2020).

Variable	Classification	Total COVID-19 patients in South Korea		
		COVID-19-Related deaths		
		Yes (N = 233)	No (N = 7,720)	Total (N = 7,953)
		N (%)	N (%)	N (%)
Avoidable hospitalizations in 2019	No	206 (2.6)	7,581 (97.4)	7,787 (100.0)
	Yes	27 (16.3)	139 (83.7)	166 (100.0)
Sex	Male	131 (4.1)	3,054 (95.9)	3,185 (100.0)
	Female	102 (2.1)	4,666 (97.9)	4,768 (100.0)
Age, years	≤69	52 (0.7)	6,889 (99.3)	6,941 (100.0)
	≥70	181 (17.9)	831 (82.1)	1,012 (100.0)
Insurance type	Medical aid	42 (6.2)	634 (93.8)	676 (100.0)
	National Health Insurance	191 (2.6)	7,086 (97.4)	7,277 (100.0)
Disability	Non-disabled	166 (2.3)	7,171 (97.7)	7,337 (100.0)
	Mild	33 (10.5)	282 (89.5)	315 (100.0)
	Severe	34 (11.3)	267 (88.7)	301 (100.0)
Underlying diseases	None	9 (0.3)	2,877 (99.7)	2,886 (100.0)
	1	18 (0.9)	1,895 (99.1)	1,913 (100.0)
	2	22 (1.9)	1,153 (98.1)	1,175 (100.0)
	≥3	184 (9.3)	1,795 (90.7)	1,979 (100.0)

COVID-19, coronavirus-19 disease.

Table 3 presents the results of the binary logistic regression analysis. In 2019, the odds ratio (OR) of death due to COVID-19 was 2.53 times higher for patients who experienced avoidable hospitalization than for their counterparts. Compared with men, women were 0.45 times more likely to die due to COVID-19. Those aged ≥ 70 years were 15 times more likely to die due to COVID-19 than those ≤ 69 years. Regarding insurance type, in the crude model, the OR of death due to COVID-19 was 2.48 times higher for health insurance than for medical aid; however, the results were not significant in the adjusted model, which controlled for the effects of other independent variables. Regarding the disability grade, the OR of death due to COVID-19 was 3.02 times higher for those with severe disability than for those without. In the case of underlying diseases, the OR of death due to COVID-19 increased as the number of underlying diseases increased compared with that of cases with no underlying disease.

**TABLE 3:** Results of logistic regression analysis (South Korea, 2019–2020).

Variable	COVID-19-related deaths			
	Total patients			
	Crude model		Adjusted model^a^	
	OR	95% CI	OR	95% CI
Avoidable hospitalizations in 2019 (Ref = No)	1.00	—	1.00	—
Yes	7.24	(4.70–11.16)	2.53	(1.52–4.20)
Sex (Ref = Male)	1.00	—	1.00	—
Female	0.51	(0.39–0.66)	0.45	(0.34–0.60)
Age (Ref ≤ 69)	1.00	—	1.00	—
≥ 70 years	28.64	(20.89–39.26)	15.00	(10.49–21.44)
Insurance type (Ref = Medical Aid)	1.00	—	1.00	—
National Health Insurance	2.48	(1.76–3.49)	1.32	(0.89–1.97)
Disability (Ref = Non-disabled)	1.00	—	1.00	—
Mild	5.11	(3.46–7.55)	1.05	(0.68–1.62)
Severe	5.56	(3.77–8.18)	3.02	(1.89–4.82)
Underlying diseases (Ref = None)	1.00	—	1.00	—
1	2.96	(1.35–6.48)	2.24	(1.01–4.95)
2	5.91	(2.76–12.65)	2.59	(1.18–5.69)
≥ 3	31.12	(16.17–59.91)	6.66	(3.32–13.39)

a Adjusted for sex, age, insurance type, disability, and underlying diseases; COVID-19, coronavirus-19 disease; OR, odds ratio; CI, confidence interval; Ref, reference.

Table 4 shows the results of the subgroup analysis. In the group aged ≥ 70 years, excluding those with underlying medical conditions, the OR of death due to COVID-19 was higher in patients who experienced avoidable hospitalization in 2019. In terms of sex, women had a lower OR for death due to COVID-19 than men in all groups. The difference in insurance type was not statistically significant. Except for people aged ≥ 70 years, the OR for death due to COVID-19 increased for patients with severe disabilities or with many underlying diseases.

**TABLE 4:** Results of logistic regression analysis for the subgroups (South Korea, 2019–2020).

Variable	COVID-19-Related deaths											
	Age group 1 ( ≤ 69 years)^a^				Age group 2 ( ≥ 70 years)^a^				Underlying disease group ( ≥1 )^b^			
	Crude model		Adjusted model		Crude model		Adjusted model		Crude model		Adjusted model	
	OR	95% CI	OR	95% CI	OR	95% CI	OR	95% CI	OR	95% CI	OR	95% CI
Avoidable hospitalizations in 2019 (Ref = No)	1.00	—	1.00	—	1.00	—	1.00	—	1.00	—	1.00	—
Yes	7.17	(2.90–17.75)	1.31	(0.49–3.51)	3.71	(2.09–6.59)	3.06	(1.69–5.54)	4.84	(3.13–7.48)	2.50	(1.51–4.15)
Sex (Ref = Male)	1.00	—	1.00	—	1.00	—	1.00	—	1.00	—	1.00	—
Female	0.23	(0.12–0.42)	0.26	(0.14–0.49)	0.58	(0.42–0.80)	0.57	(0.41–0.79)	0.48	(0.36–0.62)	0.46	(0.34–0.61)
Age (Ref ≤ 69)	—	—	—	—	—	—	—	—	1.00	—	1.00	—
≥ 70 years	—	—	—	—	—	—	—	—	18.35	(13.26–25.38)	13.13	(9.18–18.79)
Insurance type (Ref = Medical Aid)	1.00	—	1.00	—	1.00	—	1.00	—	1.00	-	1.00	-
National Health Insurance	4.48	(2.43–8.25)	0.92	(0.43–1.95)	1.35	(0.86–2.12)	1.26	(0.78–2.02)	1.88	(1.33–2.67)	1.28	(0.85–1.91)
Disability (Ref = Non-disabled)	1.00	—	1.00	—	1.00	—	1.00	—	1.00	—	1.00	—
Mild	6.36	(2.33–17.35)	2.32	(0.82–6.52)	1.04	(0.67–1.62)	0.94	(0.60–1.49)	3.61	(2.44–5.36)	1.09	(0.71–1.68)
Severe	15.99	(8.77–29.13)	6.34	(2.95–13.63)	2.04	(1.13–3.69)	1.80	(0.97–3.33)	4.09	(2.76–6.05)	3.07	(1.92–4.91)
Underlying diseases (Ref = None)	1.00	—	1.00	—	1.00	—	1.00	—	—	—	—	—
1	2.90	(0.79–10.65)	2.70	(0.74–9.84)	1.09	(0.39–3.04)	1.20	(0.43–3.34)	1.00	—	1.00	—
2	6.72	(1.93–23.40)	5.66	(1.62–16.69)	0.81	(0.30–2.17)	0.83	(0.31–2.24)	2.00	(1.08–3.71)	1.19	(0.63–2.52)
≥ 3	23.43	(7.80–70.40)	17.38	(5.71–52.96)	1.92	(0.82–4.47)	1.91	(0.81–4.49)	10.53	(6.50–17.05)	3.14	(1.87–5.28)

a Adjusted for sex, insurance type, disability, and underlying diseases.

b Adjusted for sex, age, insurance type, and disability; COVID-19, coronavirus-19 disease; OR, odds ratio; CI, confidence interval; Ref, reference.

Tables 5, 6 present the results of DRE analysis using PS. First, as shown in Table 5, after calculating IPTW, the difference between the two groups according to avoidable hospitalizations in 2019 was confirmed. As the absolute value of the standardized difference in all groups did not exceed 0.1, the difference in covariates between the two groups was not significant. Accordingly, the characteristics of the covariates in the two groups were homogeneous. Second, as shown in Table 6, the average treatment effect (ATE) in all analyses, except for the age group ≤ 69 years, confirmed that the OR of death due to COVID-19 was high for patients who experienced avoidable hospitalization.

**TABLE 5:** Differences in covariates’ standardized difference of the propensity score model (South Korea, 2019–2020).

Variable	Standardized difference							
	Total patient		Age group 1 ( ≤ 69 years)		Age group 2 ( ≥ 70 years)		Underlying disease group ( ≥ 1 year)	
	Unweighted	Weighted	Unweighted	Weighted	Unweighted	Weighted	Unweighted	Weighted
Sex (Ref = Male)	—	—	—	—	—	—	—	—
Female	−0.243	0.008	−0.276	−0.012	−0.178	−0.054	−0.285	−0.021
Age (Ref ≤ 69)	—	—	—	—	—	—	—	—
≥ 70 years	0.473	−0.009	—	—	—	—	0.317	0.037
Insurance type (Ref = Medical Aid)	—	—	—	—	—	—	—	—
National Health Insurance	−0.572	0.087	−0.718	0.088	−0.139	0.056	−0.508	0.030
Disability (Ref = Non-disabled)	—	—	—	—	—	—	—	—
Mild	0.212	−0.032	0.168	−0.038	0.028	−0.046	0.141	−0.008
Severe	0.689	−0.037	0.856	−0.045	0.221	−0.052	0.651	−0.010
Underlying diseases (Ref = None)	—	—	—	—	—	—	—	—
1	−0.285	0.015	−1.190	0.029	−0.340	-0.032	—	—
2	0.169	−0.076	0.313	−0.090	−0.223	0.066	−0.039	−0.077
≥ 3	0.860	−0.022	0.749	−0.025	0.529	0.085	0.552	0.006

Ref, reference.

**TABLE 6:** Results of doubly robust estimation analysis (South Korea, 2019–2020).

Parameter	Avoidable hospitalizations in 2019	COVID-19-Related deaths	
		OR	95% CI
Total patient group^a^	(Ref = No)	1.00	—
	Yes	1.03	(1.01–1.06)
Age group 1 ( ≤ 69 years)^b^	(Ref = No)	1.00	—
	Yes	1.00	(0.99–1.02)
Age group 2 ( ≥ 70 years)^b^	(Ref = No)	1.00	—
	Yes	1.25	(1.10–1.43)
Underlying disease group^c^	(Ref = No)	1.00	—
	Yes	1.05	(1.01–1.08)

a Adjusted for sex, age, insurance type, disability, and underlying diseases.

b Adjusted for sex, insurance type, disability, and underlying diseases.

c Adjusted for sex, age, insurance type, and disability; COVID-19, coronavirus-19 disease; OR, odds ratio; CI, confidence interval; Ref, reference.

## Discussion

### Findings

Our results indicate that the probability of COVID-19-related deaths is higher in patients who experience avoidable hospitalization.

### Interpretation

A previous study on the effect of avoidable hospitalization on mortality confirmed that the mortality rate of those who experienced avoidable hospitalization was higher than that of the general population [32]. These results are consistent with those of our study, in that those who experienced avoidable hospitalization before being diagnosed with COVID-19 were more likely to die of COVID-19 than those who did not. Patients who experienced avoidable hospitalization were unable to manage their underlying diseases well. Therefore, to reduce the death rate due to COVID-19, continuous management of existing underlying diseases is required, with preventive medicine and public health management being the most important aspects. However, medical accessibility is declining owing to social distancing, which is currently being implemented to prevent the spread of COVID-19 [33]. In addition, there is a tendency to avoid visiting medical institutions owing to the fear of contracting an infection, particularly in the case of chronically ill patients at high risk of COVID-19 and death [34]. Telemedicine may be considered as a way to reduce avoidable hospitalizations that have been occurring systematically for medical reasons during the COVID-19 pandemic. Telemedicine-based management can provide basic medical advice without exposure to the infection during a hospital visit [35], and through this, medical support such as health management, simple counseling, and medication guidance can be received [36]. The telemedicine method is one way to reduce avoidable hospitalizations that occur for personal reasons. Furthermore, this method encourages preventive self-management by providing incentives such as a reduction in out-of-pocket costs.

Attention should be paid to existing underlying and chronic diseases, appropriate preventive measures, and continuous management. In particular, because the death rate due to COVID-19 is high among people aged ≥ 70 years, pre-emptive and intensive management of elderly patients is necessary. A recent study conducted in the United States found that a number of ethnic minorities could not be hospitalized for COVID-19 due to avoidable hospitalizations among whites [37]. If hospitalization can be prevented according to primary care management, then it is possible to minimize unnecessary financial loss, promote personal health management, and prevent wastage of healthcare resources such as the need for medical personnel and facilities. Accordingly, it is crucial for hospitals to prevent avoidable hospitalizations and reduce the number of new hospitalizations so that they can effectively contribute to public health [38].

### Strengths and Limitations

The limitations of our study were as follows: First, because of limited data, it was not possible to consider the environmental characteristics of patients who died of COVID-19, such as access to medical care by region, and health behavior characteristics such as personal health habits, drinking and smoking status, and body mass index. The treatment methods could not be adjusted from patient to patient. According to the National Evidence-based Healthcare Collaborating Agency and Korean Academy of Medical Sciences’ report on COVID-19 treatment guidelines published on 12 December 2020, COVID-19 treatment guidlines were published mainly in February and March, when there were many COVID-19 confirmed cases. However, the evidence level of the guildeines was not high. The evidence level had been improved from June 2020, and COVID-19 treatment guidelines were published again in December 2020 [39]. The COVID-19 treatment methods were not fixed until July 2020 because of the earlier COVID-19 situation. Second, in the case of avoidable hospitalization, although the diseases classified in our study are different from the ACSCs suggested by the Agency for Healthcare Research and Quality, which can be seen as a limitation, it can be seen as more persuasive because they are classified according to the characteristics of Korea’s medical use. Third, because information on medical institutions’ resources cannot be included in the analysis owing to the nature of the claims data, it is difficult to distinguish whether the occurrence of avoidable hospitalization is caused by the medical system or personal factors. Fourth, there was no information on medical conditions other than the use of medical services. In particular, there is no information on the COVID-19 mutant virus, which needs to be considered because the severity and risk of death may vary depending on the mutant virus. However, because the first confirmed mutant virus in Korea occurred in December 2020 [40], this was not considered in our study and should be considered in future studies. Fifth, there may be misclassifications related to the diagnosis of ACSCs. In Korea’s health insurance system, medical institutions bill the NHI for the remaining amount, excluding the copayments paid by the patients. The Health Insurance Review and Assessment Service reviews the claims data, and the NHI pays the remaining medical expenses to the medical institution. As a result, both NHI and HIRA are strictly monitored, minimizing the possibility of misclassification [41].

Despite these limitations, our study had several strengths. First, we analyzed factors affecting the risk of death due to COVID-19, such as age and sex, insurance type, underlying diseases, disability severity, and avoidable hospitalization experience. This study also presents valuable evidence, suggesting the importance of primary care and preventive care related to avoidable hospitalization experiences and the importance of managing underlying diseases. Second, the accuracy of the results was enhanced with the use of the DRE method, with a high level of evidence for factors affecting the risk of death due to COVID-19.

### Conclusion

The effect of avoidable hospitalization experience on COVID-19-related deaths was confirmed. Therefore, continued healthcare, preventive medicine, and public health management are essential for reducing avoidable hospitalizations despite the COVID-19 pandemic. Poor disease management at a personal level causes avoidable hospitalizations and it is more likely that patients do not seek care immediately, given the context of COVID-19. There may also be an increase in COVID-19-related deaths because patients are already in poor health owing to avoidable hospitalizations. Accordingly, further research is needed on the association between COVID-19-related deaths and hospitalized patients who were not in poor health before the COVID-19 pandemic.
