# Supplementary material for: Is Avoidable Hospitalization Experienced Prior to Infection Associated With COVID-19-Related Deaths?
Source: Int J Public Health. 2022 Jun 20;67:1604426. doi: 10.3389/ijph.2022.1604426 (PMC9252312; doi:10.3389/ijph.2022.1604426)
Supplement: Supplementary file 1 [file DataSheet1.PDF]

Captions

Supplementary Figure 1. South Korea’s coronavirus disease 19 timeline(South Korea January 20, 2020 – August 14, 2020)

Supplementary Figure 2. Flow chart of the study participant selection process (South Korea 2019-2020)

Supplementary Figure 3. Directed acyclic graph (South Korea 2019-2020)

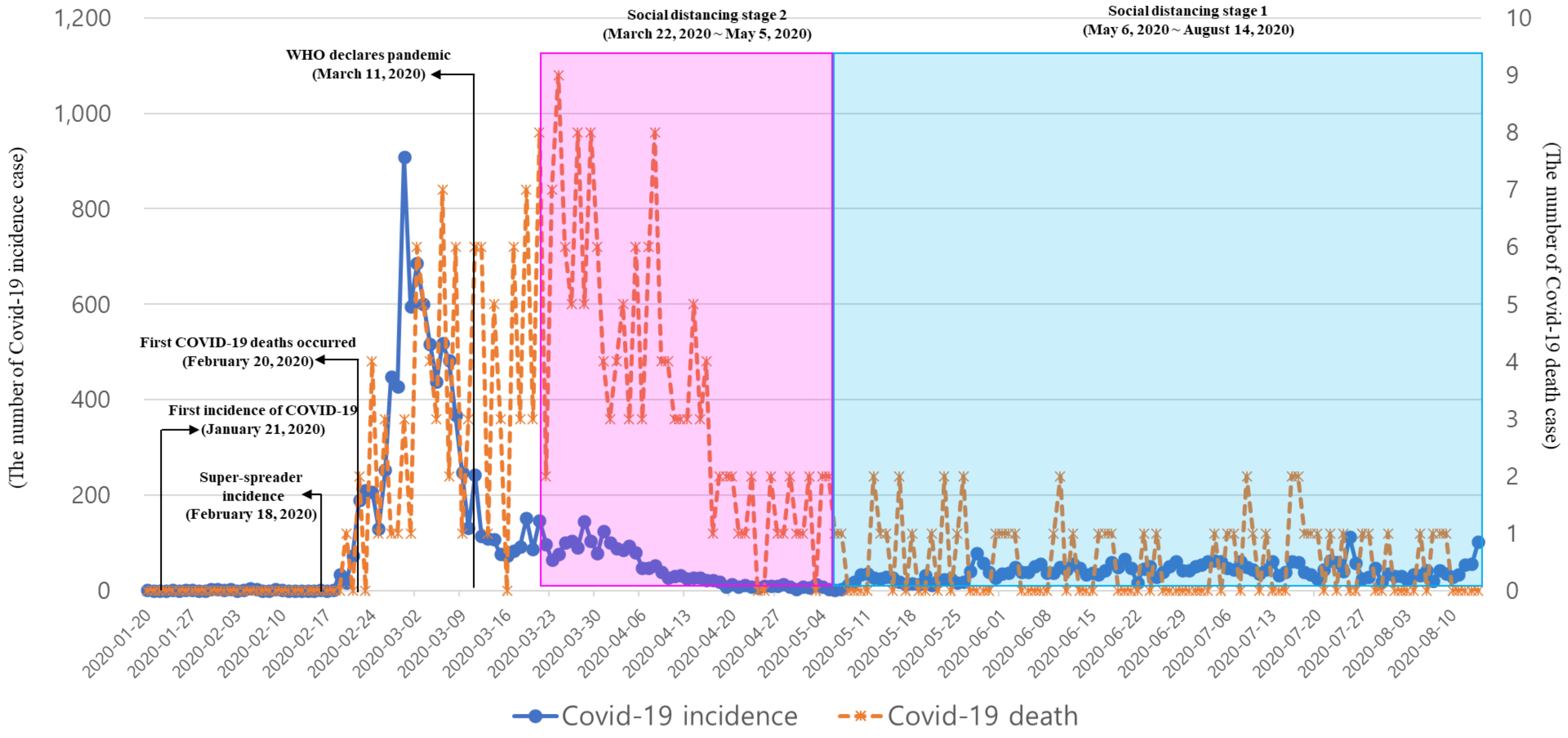

Supplementary Figure 1. South Korea's coronavirus disease 19 timeline(January 20, 2020 – August 14, 2020)

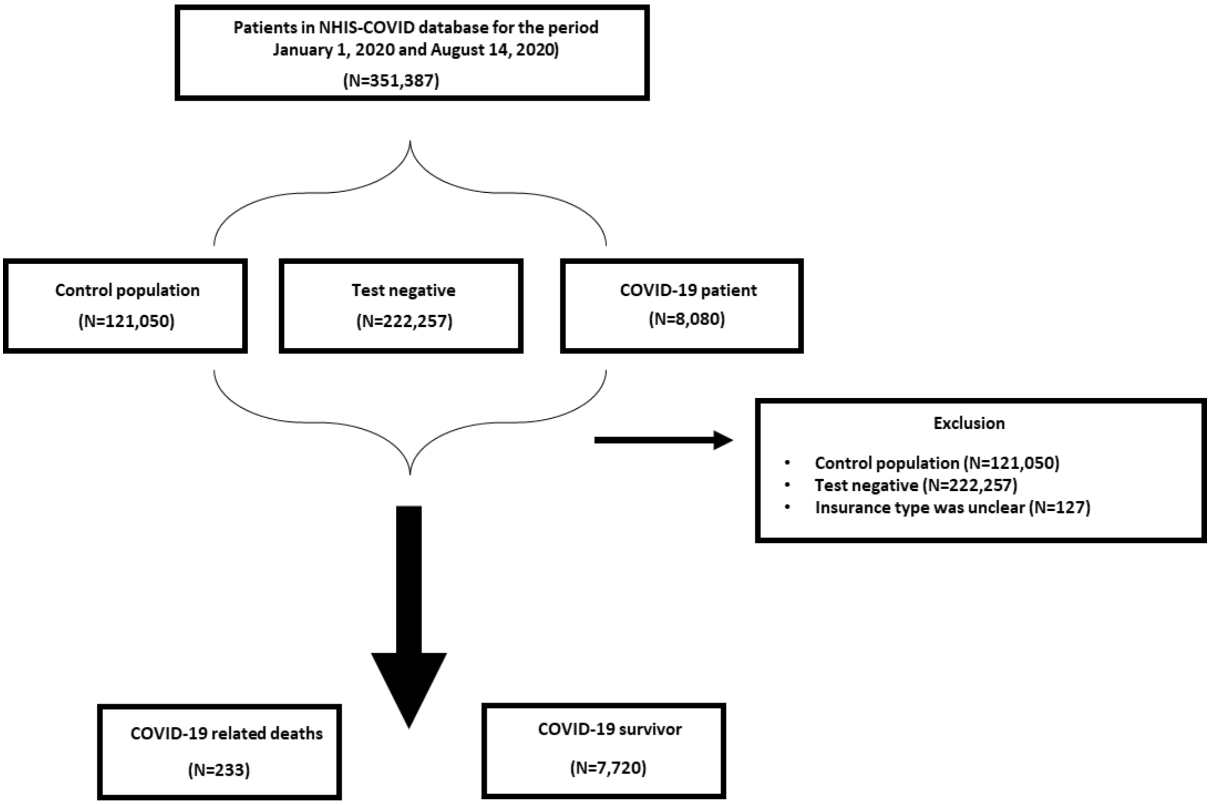

Supplementary Figure 2. Flow chart of the study participant selection process

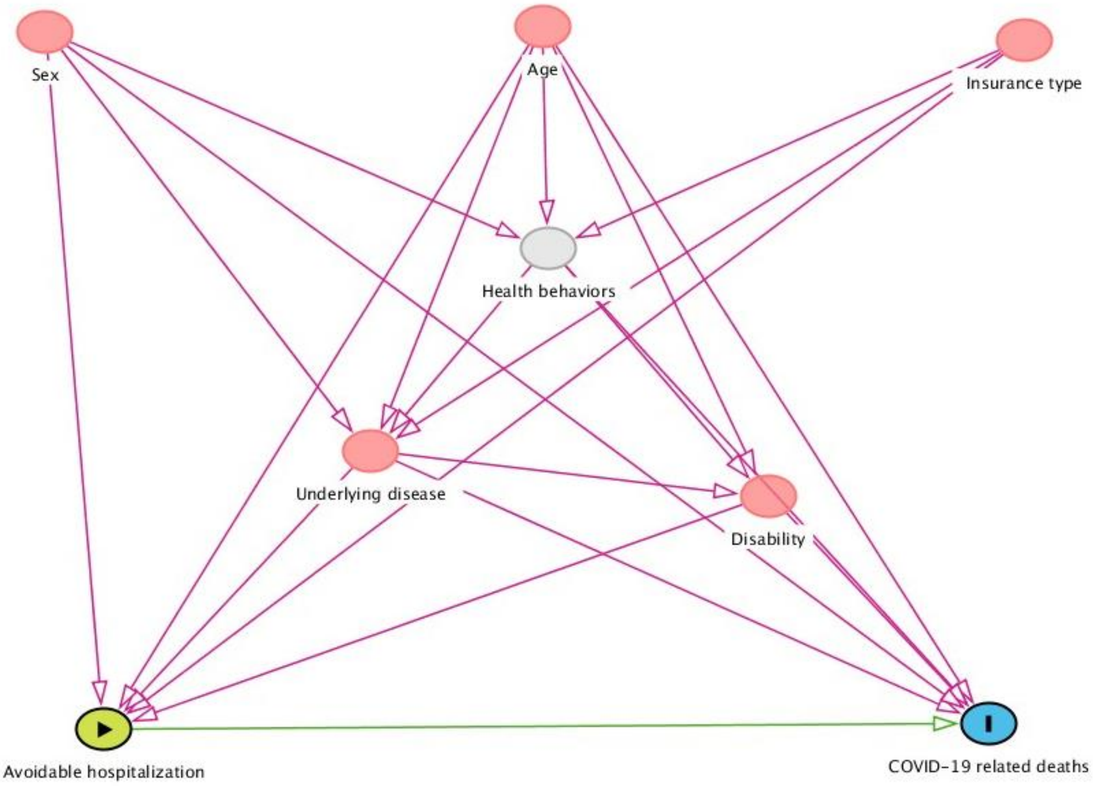

Supplementary Figure 3. Directed acyclic graph
